# A randomized trial of probiotic supplementation in nurses to reduce stress and viral illness

**DOI:** 10.1038/s41598-022-19104-9

**Published:** 2022-08-30

**Authors:** Rebecca F. Slykerman, Eileen Li

**Affiliations:** 1 Department of Psychological Medicine, University of Auckland, Building 507, 22-30 Park Avenue, Grafton, Auckland, 1023 New Zealand; 2 A Better Start – National Science Challenge University of Auckland, Auckland, New Zealand

**Keywords:** Psychology, Applied microbiology

## Abstract

Animal studies demonstrate how the gut microbiota influence psychological health and immunity to viral infections through their actions along multiple dynamic pathways in the body. Considerable interest exists in probiotics to reduce stress and illness symptoms through beneficial effects in the gut, but translating pre-clinical evidence from animal models into humans remains challenging. We conducted a large trial in nurses working during the 2020 COVID19 pandemic year to establish whether daily ingestion of the probiotic *Lactobacillus rhamnosus* HN001 reduced perceived stress and the number of days participants reported symptoms of a viral illness. Our results showed no significant difference in perceived stress or the average number of illness days between probiotic supplemented nurses and the placebo group. Stress and viral illness symptoms reduced during the study for all participants, a trajectory likely influenced by societal-level factors. The powerful effect of a well-managed public health response to the COVID19 pandemic and the elimination of COVID19 from the community in 2020 may have altered the trajectory of stress levels and reduced circulating viral infections making it difficult to detect any effect of probiotic supplementation. Our study highlights the challenge in controlling environmental factors in human trials.

## Introduction

The ubiquity of stress experienced by many people at high levels has led to an interest in novel nutritional interventions to improve psychological wellbeing. The substantial scientific advances in understanding how the microbes in the gastrointestinal tract can influence our physical and psychological health suggest that the gut microbiota is a critical factor in modulating stress^1,2^.

Dynamic communication occurs between the gut microbiota and the central nervous system along multiple physiological pathways, including the immune system, vagus nerve, parasympathetic and sympathetic arms of the autonomic nervous system and the hypothalamic–pituitary–adrenal (HPA) axis^3^. Stress increases glucocorticoid production and activation of the HPA axis, affecting immunological function and neuronal changes^3^. The production and modulation of neurotransmitters which are the chemical messaging component of the central nervous system, begin with the gut microbes as the synthesis of precursors to neurotransmitters occurs from metabolites of microbial actions^4–6^. Short-chain fatty acids, cytokines involved in neuroinflammation, and bile acids have all been researched and proposed as mechanisms explaining how the gut microbiota might influence stress^2,7^.

Against a background of pre-clinical evidence for the influence of gut microbiota on physiological processes that underpin our experience of psychological wellbeing, probiotics offer a method of manipulating the composition of the microbiota for improved health. Defined as live microorganisms that, when consumed in sufficient quantity, confer a health benefit to the host, probiotics have been studied in animal and human trials to establish their efficacy for the prevention and treatment of mental health concerns such as stress, anxiety, and depression^8,9^. In healthy volunteers, there is evidence that probiotic supplementation reduces the subjective experience of stress^10–12^. In a randomized, controlled trial in participants with moderate to high-stress levels, the probiotic *Lactobacillus plantarum* P8 reduced symptoms of stress and anxiety^13^. We have previously demonstrated that the probiotic *Lactobacillus rhamnosus* HN001 reduces postnatal depression and anxiety symptoms with the beneficial effect evident across the range of depression and anxiety scores, not restricted to only women who report high levels of symptomatology^14^.

The gut barrier protects the host from toxins and harmful antigens and plays a crucial role in healthy immunity. Psychological stress, diet, and antibiotics can alter gut barrier integrity^15^. Pre-clinical studies suggest that probiotics can improve gut barrier function in the presence of psychological stress, thereby improving immune system function^16^. In a mouse model, supplementation with *Lactobacillus rhamnosus* HN001 improved natural and acquired immune responses^17,18^. In a trial of healthy adults, low-fat milk or lactose hydrolyzed low-fat milk fortified with *Lactobacillus rhamnosus* HN001 improved systemic natural immune responses^19^. In a small study of patients with irritable bowel syndrome (IBS), supplementation with a combination of *Lactobacillus rhamnosus* HN001, *Bifidobacterium longum* BB536, and vitamin B6 improved gut permeability and microbial composition as well as reduced the symptoms of IBS^20^. At a population level, \probiotics can reduce the incidence, symptom duration and related absence from work or school of viral illnesses in healthy adults and children^21–23^.

Nurses encounter increased exposure to viral illnesses at work and experience stress at a higher level than the general population due partly to the demands of caring for patients and families who are themselves, at a stressful point in their lives and the high workload in many health settings. The COVID19 pandemic introduced a series of novel stressors across the population, particularly for nurses working in clinical roles. Higher rates of anxiety, stress, fear, and burnout have been reported in surveys of nurses conducted early in the 2020 pandemic year^24–26^.

This study aimed to investigate whether supplementation with the probiotic *Lactobacillus rhamnosus* HN001 could reduce symptoms of stress and anxiety and improve psychological wellbeing in nurses working during the COVID19 pandemic. A further aim was to determine whether supplementation with *Lactobacillus rhamnosus* HN001 could reduce the number of days participants experienced symptoms of a viral illness.

## Results

**Figure 1:**
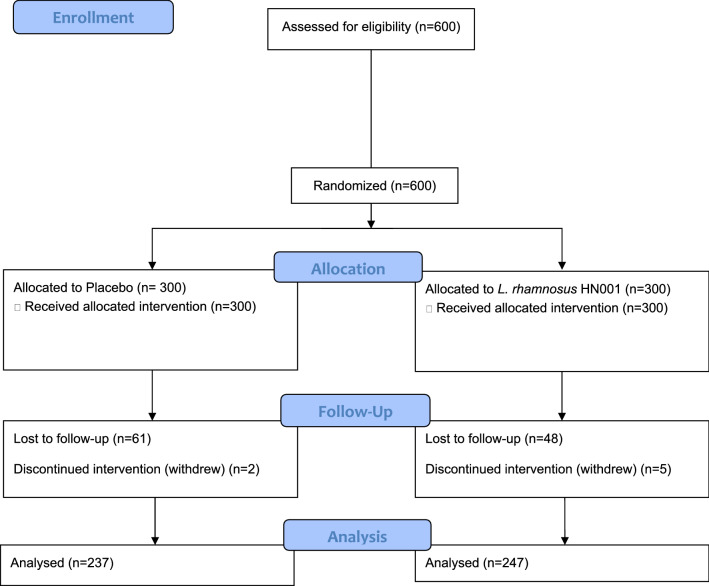
CONSORT Flow diagram for the recruitment and randomization of participants.

Of the 600 nurses enrolled in the trial, 484 (80.7%) completed the end of intervention questions. Figure 1 shows the CONSORT flow diagram for the trial. There were no significant differences between respondents and non-respondents to the end of intervention questions in the intervention group (*p* = 0.85), sex (*p* = 0.61), baseline anxiety (*p* = 0.26), or baseline wellbeing scores (*p* = 0.59). Younger nurses aged 18–24 years (*p* = 0.01) and Asian nurses (*p* < 0.0001) were less likely to respond at follow up, and non-respondents had significantly higher baseline stress scores than respondents (*p* = 0.01).

Table 1 shows the sample characteristics. There were no significant differences between the probiotic supplemented group and the placebo group in demographic factors or measures of psychological health at baseline.

**Table 1:** Sample characteristics for the probiotic and placebo groups.

	Placebo	HN001
	N (%)	N (%)
**Sex**		
Male	11 (3.67)	8 (2.67)
Female	289 (96.33)	292 (97.33)
**Ethnicity**		
European	218 (72.67)	228 (76.00)
Māori	20 (6.67)	22 (7.33)
Pacific	6 (2.00)	3 (1.00)
Asian	22 (7.33)	16 (5.33)
Other	34 (11.33)	31 (10.33)
**Age group**		
18–24	35 (11.67)	33 (11.00)
25–34	89 (29.67)	95 (31.67)
35–44	54 (18.00)	61 (20.33)
45–54	69 (23.00)	63 (21.00)
55–70	53 (17.67)	48 (16.00)
**Place of work**		
Hospital	196 (65.33)	208 (69.33)
GP	30 (10.00)	35 (11.67)
Community	50 (16.67)	32 (10.67)
Residential	6 (2.00)	9 (3.00)
Other	14 (4.67)	14 (4.67)
Missing	4 (1.33)	2 (0.67)
**Hours/week worked**		
5–10	1 (0.33)	3 (1.00)
11–20	13 (4.33)	13 (4.33)
21–30	39 (13.00)	55 (18.33)
31–40	197 (65.67)	196 (65.33)
41 +	50 (16.67)	33 (11.00)

Baseline scores (n = 600)	Mean (SD) n = 300	Mean (SD) n = 300
Stress	19.1 (5.5)	19.1 (5.2)
Anxiety	57.2 (12.0)	56.7 (12.8)
Wellbeing	11.8 (3.9)	11.9 (4.1)

Post intervention scores (n = 484)	Mean (SD) n = 237	Mean (SD) n = 247
Stress	14.7 (6.1)	14.7 (5.8)
Anxiety	63.7 (12.7)	62.7 (12.1)
Wellbeing	14.6 (4.5)	14.6 (4.3)

Change in stress, anxiety, and psychological wellbeing between baseline and the end of intervention did not significantly differ between the probiotic and placebo groups (Table 2).

**Table 2:** Mean change in stress, anxiety, and psychological wellbeing for the probiotic and placebo groups.

	Placebo	HN001	*p*-value 0.78
	N = 237	N = 247	
	Mean change in score (SD)		
Stress	4.1 (5.7)	4.2 (5.4)	0.78
Anxiety	− 5.7 (12.8)	− 6.0 (13.0)	0.82
Wellbeing	− 2.7 (4.2)	− 2.6 (4.0)	0.83

### Effect of treatment group on average illness days

The number of days with symptoms of illness in week 1 of the intervention period was a measure of baseline illness. It did not differ significantly between the probiotic and placebo groups (*p* = 0.44). The average number of illness days per week over the intervention period (weeks 2–12) did not differ between the probiotic and placebo groups (Table 3).

**Table 3:** Mean illness days per week for weeks 2–12 of the trial for the probiotic and placebo groups.

Treatment	N	Mean	SD	*P*-value
Placebo	252	0.83	1.64	0.59
HN001	273	0.91	1.69	

**Figure 2:**
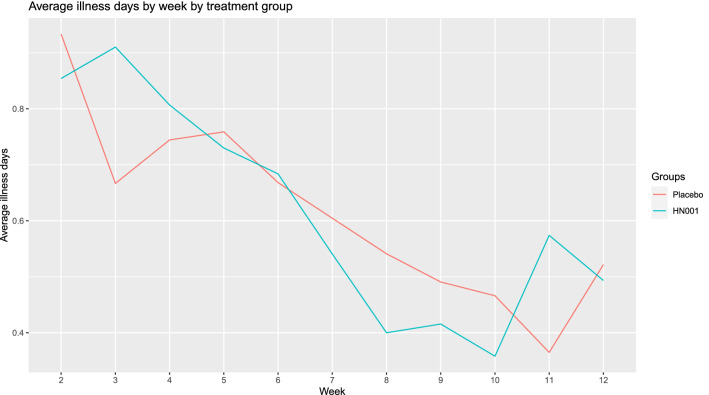
Average illness days per week for weeks 2–12 of the trial for the probiotic and placebo groups.

Figure 2 shows the average number of illness days per week by intervention week for the probiotic and placebo groups. It shows the decrease in average illness days per week over the intervention period and graphically demonstrates that this decrease occurred in both the placebo and probiotic groups.

### Effect of time on average illness days per week

There was a significant time effect on the average number of illness days per week for the study cohort. As time increased, the average number of illness days per week decreased (*p* = 0.0004) (Table 4). There was no significant interaction between time and treatment group for average illness days per week (*p* = 0.37).

**Table 4:** Mean illness days per week of intervention.

Week of intervention	Sample size	Mean (SD)
2	517	0.85 (1.69)
3	498	0.78 (1.02)
4	492	0.75 (1.57)
5	489	0.73 (1.60)
6	469	0.66 (1.46)
7	467	0.57 (1.28)
8	445	0.46 (1.27)
9	445	0.45
10	459	0.42
11	461	0.47
12	438	0.50

**Figure 3:**
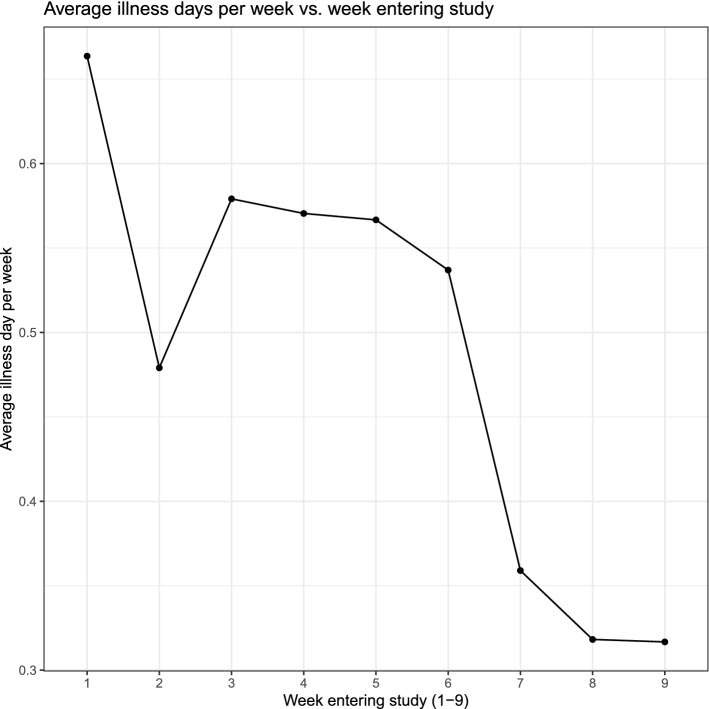
Average illness days per week by week of entering the study.

Figure 3 shows the average number of illness days per week by week of entry into the study.

Similar to the observed time effect on reducing illness days per week, later entry into the study was associated with a lower number of average illness days per week (β − 0.41 95%CI: − 0.75, − 0.08; *p* = 0.01).

## Discussion

We conducted a large randomized controlled trial to test the effectiveness of the probiotic *Lactobacillus rhamnosus* HN001 for improving psychological health and reducing average illness days per week in nurses working during the 2020 COVID19 pandemic year. Following the intervention, stress, anxiety, and psychological wellbeing were not significantly different between nurses supplemented with the probiotic and those who received the placebo.

Stress and psychological wellbeing improved from baseline to the end of the intervention period, a trajectory that may not represent the typical pattern for nurses working during 2020 in other countries. New Zealand was one of only a few countries to successfully eliminate COVID19 from the community in 2020 using lockdown measures and border controls. Following a strict government-led lockdown from 25 March to 13 May 2020, New Zealanders had relatively few restrictions on daily life, and there were no community cases of COVID19. We conducted this study in the second half of 2020. During the study, a brief two-week lockdown of Auckland, the largest metropolitan city in New Zealand, was followed by a period of few restrictions and a nationwide move to Alert Level 1, which exerts no restrictions apart from limiting international travel. Confidence in government and public health measures to manage COVID19 and, therefore, fewer pressures in the health system than seen in other countries may be the reason for the observed improvement in the psychological health of nurses in this study. The powerful environmental effects of a well-managed COVID19 response may have led to a pattern of decrease in stress that swamped any benefit of probiotics. A recent review considering the challenges of translating pre-clinical evidence into interventions for mental health in humans acknowledges the significant complexity of phenomena such as stress, anxiety, or depression. These psychological states have dynamic contributors, including macro-level events in society that researchers cannot control in human studies as in animal models^27^.

The other important finding to emerge from this clinical trial is that the average number of days per week that nurses reported symptoms of cold or flu-like illness did not significantly differ between the probiotic and placebo supplemented groups. The number of circulating viral illnesses during 2020 in New Zealand significantly decreased^28^. Hand hygiene, awareness of social distancing, particularly from those with a viral illness, and strict border closures are likely to have resulted in fewer viruses circulating in the community in 2020. With less exposure to illness and therefore fewer days of symptoms across the population, it may have been more difficult to detect a beneficial effect of probiotic supplementation on viral illness symptoms.

Probiotic manipulation of the gut microbiota is known to be strain-specific. An alternative strain of probiotics may have enhanced psychological health and reduced illness days. However, we have previously demonstrated a significant and positive effect of supplementation with *Lactobacillus rhamnosus* HN001 on postnatal depression and anxiety, suggesting the HN001 strain is associated with improved psychological health. In that study, the positive effect of probiotic supplementation was present for participants with depression and anxiety scores across the spectrum and not restricted to those reporting high levels of distress^14^. Associated improvement in immune markers has been demonstrated using the HN001 strain in animal studies^17,18^ and human trials^19^.

This study was conducted in healthy volunteers and not in a clinical population with diagnoses of mental health problems. Nurses who did not respond at the end of intervention had higher baseline stress scores than those who responded. It is possible that the probiotic did not exert a beneficial effect on mood in this sample of healthy nurses. However, we have previously shown that *Lactobacillus rhamnosus* HN001 decreased postnatal depression and anxiety scores across the range of symptom scores, and the effect was not restricted to women with high symptomatology^14^. Secondly, the stress scores of participants in our study were higher than average at baseline, and 84.3% of nurses reported stress levels in the moderate to high range using established cut-offs.

There are some limitations of our study that require discussion. We did not assess participants' gut microbiota or measure physiological markers of either immune function or psychological stress. Therefore, we cannot discuss the physiological profile of participants according to the intervention group. The exact mechanism by which probiotics improve psychological health is likely to be strain-specific and has not been fully elucidated. Alterations in response to stress and increased diversity in the gut microbiota have been associated with *Lactobacillus casei* strain Shirota supplementation^29^. However, a review of randomized, controlled trials in healthy volunteers found no consistent evidence for an effect of probiotics on cortisol^10^. Night shift nurses supplemented with yoghurt containing probiotics had improved markers of immune function; however, the study did not measure symptoms of viral illness, and it is unclear whether changes in immune markers are observable in illness symptomatology^30^.

Adherence to daily capsule intake may have contributed to the lack of a significant difference between groups in our study. All data collection and study processes used an online platform, and participants did not attend any in-person appointments during which we could ascertain capsule numbers. Automated electronic monitoring was beyond the scope of this trial. However, participants in this trial were nurses working in clinical settings, a population who would be expected to adhere to the daily intake of capsules. Participants had regular contact with the researchers and received weekly text messages throughout the intervention. Furthermore, we had a high post-intervention retention rate indicating a commitment to participation in the trial. We used an intent to treat analysis as an appropriate approach to data collected in a real-world setting. The strengths of our study include the large sample size and high post-intervention retention rate of participants.

In conclusion, we conducted a large double-blind, placebo-controlled trial of probiotic supplementation to reduce stress and days of illness symptoms in nurses, we found no significant difference in outcomes between the probiotic and placebo groups. A well managed public health response to the COVID19 pandemic is likely to have altered the trajectory of stress in this study population. Furthermore, fewer circulating viral illnesses during 2020 impacted the potential to detect a beneficial effect of probiotics on viral symptoms. These wider contextual events highlight one of the challenges of conducting intervention trials in human populations.

## Methods

The study was a randomized, double-blind, placebo-controlled trial with two parallel arms and a ratio of allocation to probiotic or placebo of 1:1.

### Participants

Eligible participants were registered nurses working in a clinical environment anywhere in New Zealand, aged between 18 and 70 years old. Exclusion criteria were: taking a regular probiotic supplement, currently taking immunosuppressant, e.g. chemotherapy, or taking part in another research trial. Participating nurses enrolled in the study between 2 July 2020 and 26 August 2020.

### Data collection

All consent and data collection were managed through a secure online web interface, and participants were not required to attend any in-person appointments. Nurses enrolled in the study and completed the questionnaires using their mobile phone, tablet or computer.

### Randomization

Following enrollment in the study, participants were randomized to receive the probiotic or placebo. As nurses completed baseline consent and registration information, they were assigned the following available sequential study number and provided the corresponding bottle of capsules. Fonterra Cooperative Group Limited managed the randomization schedule containing study numbers and intervention group, and Fonterra was not involved with the recruitment, collection of data or analysis of results. Both participants and the researchers were blind to the randomization schedule.

### Intervention

Probiotic capsules containing *Lactobacillus rhamnosus* HN001 (6 × 10^9^ colony forming units) and placebo capsules identical in appearance and smell to the probiotic-containing corn-derived maltodextrin were supplied by Fonterra Cooperative Group Limited. All capsules were manufactured to pharmaceutical grade and were lactose-free and gluten-free. Studies conducted in New Zealand have safely given the probiotic *Lactobacillus rhamnosus* HN001 (6 × 10^9^ CFU) to pregnant women and infants^14,31^.

Nurses were instructed to take one capsule a day for 12 weeks from when they enrolled in the study and were provided with capsules. Participants entered the study in nine waves with a weekly cut-off that determined the wave of participants for that week. Therefore, a weekly delivery of capsules to the participants who enrolled in the study each week ensured that each wave of participants began the intervention simultaneously.

### Measures

Nurses provided demographic and employment information at the time of enrolment into the study.

Age was recorded in years in one of five categories (18–24, 25–34, 35–44, 45–54, 55–70 +).

In New Zealand, it is common for people to identify with more than one ethnic group. A system of prioritized ethnicity is used in these cases. Individuals who list more than one ethnic group are assigned to one according to the following order of priority: Māori, Pacific, Asian, European, MELAA (Middle Eastern, Latin American or African) and others.

### Employment information

The typical number of hours worked per week was recorded as 1–10, 11–20, 21–30, 31–40 or 41 + .

Nurses identified their primary work setting by choosing one of five groups (Hospital, General Practice/Family Doctor, Community Service, Residential Care Facility or other).

### Outcome measures

#### Psychological health outcomes

Nurses completed psychological health questionnaires at baseline and again at the end of the 12 week intervention period.

#### Stress

The primary outcome measure for this trial was perceived stress. The Perceived Stress Scale is a 10 item questionnaire that asks about stress and coping in the previous month. Scores range from 0 to 40, with higher scores indicative of higher stress^32^.

#### Anxiety

State-Trait Anxiety Inventory 6 item version (STAI6): The STAI6 is a short 6 item scale validated as an anxiety screening questionnaire based on the more extended State-Trait Anxiety Inventory^33^.

#### Psychological well-being

The World Health Organisation—Five Well-Being Index (WHO-5) is a five-item, positively worded measure of psychological wellbeing with scores ranging from 0 to 25. Higher scores represent higher levels of wellbeing. A systematic review of the WHO-5 concluded that it was a widely used and sensitive measure of depression^34^.

#### Illness

Each week on a Monday, participants received a text message asking them to reply to a single question: “How many days this week (since last Monday) have you had symptoms of cold/flu (0–7)? Please reply to this message. Each participant received a total of 12 text messages, one for each week of the intervention period.

For each of the 12 weeks, the average number of illness days per week was calculated. The first week was a measure of pre-intervention illness. For the remaining 11 weeks, an average was calculated for the period, for the probiotic and placebo groups, and the total sample.

### Sample size

An initial sample size calculation aimed to recruit a total of 507 participants to give a final post-attrition sample size of 380 allowing 90% power to detect a decrease of one-third of a standard deviation in stress score (effect size 3 points on the PSS), at the 5% level of significance. The calculation was based on a previous study of stress in healthcare workers that used the Perceived Stress Scale (mean = 40.9, SD = 8.9)^35^.

The actual sample size achieved was 484 (post-attrition), which gave a 90% chance of detecting a difference in stress scores of 0.3 standard deviations (effect size 1.768) between the probiotic and placebo groups at the 5% level of significance in our sample.

### Statistical analysis

Intent-to-treat analysis was conducted in SAS 9.4 using a two-sample t-test. Change in stress, anxiety and psychological wellbeing was calculated by subtracting post-intervention scores from baseline scores for each of the three measures. The findings are reported according to the CONSORT statement.

### Analysis of illness data

#### Effect of treatment group on illness days

We examined the association between intervention group allocation and average illness days by comparing the mean illness days for each group using a two-sample t-test.

#### Time effect

A repeated measures analysis examined the relationship between time (week of intervention), group allocation (probiotic or placebo) and the average number of illness days per week.

Participants enrolled in the study over nine weeks. Bivariate regression analysis examined the association between week entering the study and average illness days (weeks 2–12. The average number of illness days per week for the study was plotted by the week of entry (1–9 weeks).

### Ethical approval

The study received full ethical approval from the Auckland Health Research Ethics Committee (Reference AH1335) and was conducted in accordance with all relevant guidelines and regulations. All participants gave informed consent for their participation. The trial was registered with the Australian New Zealand Clinical Trials Registry on16/04/2020. Registration number ACTRN12620000480987.

## Supplementary Information

Supplementary Information.

## Data Availability

Raw data from which these results are derived is available as supplementary material.
